# In vitro anti-HIV activity of some Indian medicinal plant extracts

**DOI:** 10.1186/s12906-020-2816-x

**Published:** 2020-03-06

**Authors:** Aparna Palshetkar, Navin Pathare, Nutan Jadhav, Megha Pawar, Ashish Wadhwani, Smita Kulkarni, Kamalinder K. Singh

**Affiliations:** 1 C. U Shah College of Pharmacy, S.N.D.T. Women’s University, Santacruz West, Mumbai, 400049 India; 2 National AIDS Research Institute, 73, ‘G’-Block, MIDC, Bhosari, Pune, 411 026 India; 3 School of Pharmacy and Biomedical Sciences, Faculty of Clinical and Biomedical Sciences, University of Central Lancashire, Preston, PR1 2HE UK

**Keywords:** HIV, TZM-b1, PM1, *Achyranthes aspera*, *Rosa centifolia*

## Abstract

**Background:**

Human Immunodeficiency Virus (HIV) persists to be a significant public health issue worldwide. The current strategy for the treatment of HIV infection, Highly Active Antiretroviral Therapy (HAART), has reduced deaths from AIDS related disease, but it can be an expensive regime for the underdeveloped and developing countries where the supply of drugs is scarce and often not well tolerated, especially in persons undergoing long term treatment. The present therapy also has limitations of development of multidrug resistance, thus there is a need for the discovery of novel anti-HIV compounds from plants as a potential alternative in combating HIV disease.

**Methods:**

Ten Indian medicinal plants were tested for entry and replication inhibition against laboratory adapted strains HIV-1_IIIB_, HIV-1_Ada5_ and primary isolates HIV-1_UG070_, HIV-1_VB59_ in TZM-bl cell lines and primary isolates HIV-1_UG070_, HIV-1_VB59_ in PM1 cell lines. The plant extracts were further evaluated for toxicity in HEC-1A epithelial cell lines by transwell epithelial model.

**Results:**

The methanolic extracts of *Achyranthes aspera, Rosa centifolia* and aqueous extract of *Ficus benghalensis* inhibited laboratory adapted HIV-1 strains (IC_80_ 3.6–118 μg/ml) and primary isolates (IC_80_ 4.8–156 μg/ml) in TZM-bl cells. Methanolic extract of *Strychnos potatorum*, aqueous extract of *Ficus infectoria* and hydroalcoholic extract of *Annona squamosa* inhibited laboratory adapted HIV-1 strains (IC_80_ 4.24–125 μg/ml) and primary isolates (IC_80_ 18–156 μg/ml) in TZM-bl cells. Methanolic extracts of *Achyranthes aspera and Rosa centifolia,* (IC_80_1-9 μg/ml) further significantly inhibited HIV-1 primary isolates in PM1cells. Methanolic extracts of *Tridax procumbens, Mallotus philippinensis, Annona reticulate,* aqueous extract of *Ficus benghalensis* and hydroalcoholic extract of *Albizzia lebbeck* did not exhibit anti-HIV activity in all the tested strains. Methanolic extract of *Rosa centifolia* also demonstrated to be non-toxic to HEC-1A epithelial cells and maintained epithelial integrity (at 500 μg/ml) when tested in transwell dual-chamber.

**Conclusion:**

These active methanolic extracts of *Achyranthes aspera* and *Rosa centifolia,* could be further subjected to chemical analysis to investigate the active moiety responsible for the anti-HIV activity. Methanolic extract of *Rosa centifolia* was found to be well tolerated maintaining the epithelial integrity of HEC-1A cells in vitro and thus has potential for investigating it further as candidate microbicide.

## Background

Human Immunodeficiency Virus (HIV) persists to be a significant public health issue worldwide. In 2018, 37.9 million people are living with HIV globally; out of which 36.2 million are adults and 1.7 million are children less than 15 years old. There were 1.7 million new infections and 770,000 people died from AIDS related illness worldwide [1]. The current strategy for the treatment of HIV infection is Highly Active Antiretroviral Therapy (HAART), based on combination of inhibitors of reverse transcriptase and protease. Although HAART has considerably reduced deaths from AIDS related disease, it often has side effects and not well tolerated especially in persons undergoing long term treatment and maintains the risk of developing multidrug resistance [2]. Moreover, HAART is an expensive regime for underdeveloped and developing countries where the drugs are inaccessible to the HIV infected patients. Thus, there is a need for the discovery of novel therapeutic strategies, which identify new anti-HIV compounds from natural sources particularly from medicinal plants.

Natural sources provide a large reservoir for screening of anti-HIV agents with novel structure and antiviral mechanism due to their structural diversity. For the purpose of this study, ten Indian traditional medicinal plants, *Albizzia lebbeck*, *Tridax procumbens, Achyranthes aspera*, *Ficus benghalensis*, *Mallotus philippinensis*, *Rosa centifolia, Strychnos potatorum*, *Annona reticulate*, *Ficus infectoria* and *Annona squamosa* were selected to investigate their in vitro inhibitory activity against entry inhibition/replication of HIV-1 as first step towards identification of potential anti-HIV microbicide. The microbicides provide protection by directly inactivating HIV or preventing HIV from attaching, entering or replicating in susceptible target cells as well as dissemination from target cells present in semen or the host cells that line the vaginal/rectal wall [3]. These plants were selected on the basis of detailed patent survey and scientific articles on the ethnomedicinal usages of the plant genera directly in HIV/AIDS or for symptoms/conditions closely associated with this disease (Table 1).

**Table 1:** Ethnomedicinal usages of selected plant materials

Sr No	Botanical name	Common name	Family	Conventional use and published reports
1	*Albizzia lebbeck*	Shirisha	Mimosaceae	Bark: Anti-oxidant, anti-fertility, anti- microbial activity Seeds: Anti-inflammatory activity [4, 5].
2	*Tridax procumbens*	Ghamra	Asteraceae	Whole plant: Anti-microbial Flowers, Leaves: Anti septic, insecticidal, parasiticidal, anti-Cancer Activity Aerial parts: Hepatoprotective Leaves: Hypotensive, anti- diabetic, immunomodulating activity [6–12].
3	*Achyranthes aspera*	Apaamaarga	Amaranthaceae	Whole plant: Nephroprotective, hypolepidemic activity. Roots: anti-oxidant, spermicidal, activity Leaves: anti-oxidant, anti-fertility, anti-depressant, anti-cancer, anti- microbial activity Aerial parts: Hepatoprotective activity Seeds: Anti- microbial activity [13–22].
4	*Ficus benghalensis*	Vad	Moraceae	Whole plant: anthelmentic, anti-bacterial activity. Bark: Anti-inflammatory, anti-bacterial activity. Aerial roots: anti-oxidant, anti-diabetic, immunomodulatory activity [23–27].
5	*Ficus infectoria*	Pilkhan		Bark and Leaves: anti-oxidant, anti-hyperlipidemic, hypoglycemic activity [28].
6	*Mallotus philippinensis*	Kamala	Euphorbiaceae	Seeds: Anti-fertility activity. Stembark: anti-oxidant, anti-tumor activity,anti- bacterial. Fruits: anti-inflammatory, immunoregulatory, anti-proliferative activity. Leaves: Hepatoprotective activity Roots: Anti-leukaemic activity [29–36].
7	*Rosa centifolia*	Gulab	Rosaceae	Leaves: treating wounds, ophthalmia, hepatopathy, hemorrhoids and anti-microbial, Flowers: cardio tonic, anti-inflammatory, anti-asthmatic, anti-bronchitic, anti-diarrheal, dysmenorrheal, urinary tract infections, anti-tussive activity [37, 38].
8	*Strychnos potatorum*	Nirmali	Loganiaceae	Plant: Anti-diabetic, anti- microbial activity Seeds: Contraceptive, diuretic, anti-inflammatory, hepatoprotective, antioxidant, antiarthritic activity [39–45].
9	*Annona reticulate*	Ramphal	Annonaceae	Leaves: Anti-oxidant, anti- inflammatory, anti-helmentic activity. Seeds: Anti-cancer [46–48].
10	*Annona squamosa*	Sitafal		Bark: Anti-malarial activity Seeds: Anti-tumor activity Twigs: Anti-ulcer activity Leaves: anti- oxidant,hepatoprotective, anti- bacterial activity Fruit pulp: Anti-HIV activity [49–55].

Legend: Details of plants selected and their reported conventional use

Plants such as *R. centifolia, S. potatorum, F. infectoria*, *F. benghalensis* and *M. philippinensis* were selected because other species of the same genera have exhibited anti-HIV activity [56–60]. Its traditional use in gonorrhoea and leukeorrhea [61] and suppressive effects on sperm motility [39] further made *S. potatorum,* a plant of choice for this study. Fruit pulp of *A. squamosa* has been reported to inhibit HIV replication significantly in H9 lymphocytes [49] therefore the seeds of *A. squamosa* which have also shown spermicidal property, an additional desirable attribute for a vaginal microbicide [62] was selected for the study. In addition the leaves of other species *A. reticulate* were also selected for assessing the anti-HIV activity.

Taylor et al., [63, 64] reported methanolic extract of *T. procumbens* to exhibit in-vitro anti-Herpes Simplex Virus activity in Vero cells; hence it was selected for investigating its anti-HIV activity. Anticipating the potential of spermicide-based vaginal contraceptives in the reproductive health of women such as Nonoxynol (N-9) and Praneem polyherbal (*Azadirachta indica* leaves, *Sapindus mukerossi* pericarp of fruit and *Mentha citrate* oil) [65]; two plant extracts, methanolic leaf extract of *A. aspera* that has exhibited safety as well as good anti-fertility property [66] and methanolic pod extract of *A. lebbeck* which has been shown to suppress spermatogenesis and alter the structure and activity of the Sertoli and Leydig cells [4] were considered worthwhile to explore for anti-HIV activity.

Therefore, under the DBT-ICMR sponsored programme (HIV/AIDS and Microbicides, Phase I) developed for screening plant derived HIV microbicidal candidates, we evaluated these 10 plant extracts against 2 CXCR4 (HIV-1_IIIB_, HIV-1_UG070_) and 2 CCR5 tropic (HIV-1_Ada5_, HIV-1_VB59_) HIV-1 strains.

## Methods

### Plant materials and extraction

10 plant materials were collected from various parts of India in different seasons. A plant taxonomist at publicly available herbarium, Botanical Survey of India, Pune, India, validated scientific names and classification of these plants. The specimens were also deposited in the herbarium. Table 2 presents ethno-botanical information and solvents used for extraction of the selected plants.

**Table 2:** Procurement, authentication and solvents used for extraction of plant material

Sr. No.	Plant Name	Part of plant	Authentication No.	Solvent for extraction	% yield (± SD)
1	*Albizzia lebbeck*	Whole pods	RR 3794	Hydroalcohol	17.20 (±0.32)
2	*Tridax procumbens*	Aerial parts	KK1	Methanol	7.2 (±1.32)
3	*Achyranthes aspera*	Aerial parts	PADAAP1	Methanol	10.74 (±0.02)
4	*Ficus benghalensis*	Leaves	PADFB1	Water	7.56 (±0.52)
5	*Mallotus philippinensis*	Leaves	MPADP12	Methanol	5.72 (± 0.09)
6	*Rosa centifolia*	Leaves	ROAP1	Methanol	9.46 (± 0.56)
7	*Strychnos potatorum*	Seeds	SPAP2	Methanol	15.00 (±0.43)
8	*Annona reticulata*	Leaves	APAR1	Methanol	7.89 (±0.07)
9	*Ficus infectoria*	Leaves	APF1	Water	19.08 (±0.02)
10	*Annona squamosa*	Seeds	SS1/ 2008	Hydroalcohol	10.87 (±0.15)

Legend: Procurement, authentication no. & extraction details of the plants parts used for the study

The collected plant materials were cleaned, freed of foreign contaminates and washed with water, first air dried and then dried in an electric oven at 40 °C. The dried plant materials were pulverized in an electric mixer. The plant materials were extracted with various solvents individually by hot continuous Soxhlet extraction method for 18–24 h. After extraction, the extract obtained was filtered through 0.2-μm syringe filter and then concentrated on a rotary evaporator by distilling off the solvent under vacuum at 40 °C. The concentrated extracts was finally lyophilized to obtain free flowing powder and stored in airtight bottles in the refrigerator at 4-8 °C. The extractive yields of the individual extracts are recorded in Table 2. Powder was reconstituted in DMSO for final concentration of extract 10 mg/ml and stored at -20 °C until tested for anti-HIV1 activity.

### Preliminary phytochemical investigation

Qualitative tests were carried out to ascertain the presence of various phytochemicals in the plants extract of the selected plants using the methods described by Harbourne [67] (Table 3). It involved the appropriate addition of chemicals and reagents to the concentrated extract of the plant material in a test tube. The changes in the appearance of the colour, as the case may be, confirmed the presence of alkaloids, flavanoids, tannins, steroids and saponins.

**Table 3:** Phytochemical screening of selected plant extracts

Sr. No.	Plant Extracts	Steriods	Saponins	Flavanoids	Alkaloids	Tannins/ Phenolic Compounds
1	*A. lebbeck*	+++	+	+	++++	++
2	*T. procumbens*	–	–	+	–	–
3	*A. aspera*	+++	+	+	++++	++++
4	*F. benghalensis*	+++	+	+	++++	+++
5	*M. philippinensis*	–	+	+	++++	++
6	*R. centifolia*	–	+	+	++++	++
7	*S. potaotorum*	+++	+	–	++++	++
8	*A. reticulate*	+++	–	+	++++	+++
9	*F. infectoria*	++	+	++	++++	++++
10	*A. squamosa*	–	+	+	++++	++

Legend: presence or absence of phytochemical components in plant extracts by different methods

-: Absent, ++++: Present in large proportion, +++: Present in good proportion, ++: Present in moderate proportion, +: Present in low proportion

Tests for Steroid: 1. Salkowski Reaction 2. Liebermann- Burchard Reaction 3. Liebermann’s Reaction

Tests for Saponins: 1. Foam Test

Tests for Flavanoids: 1. Shinoda Test 2. Lead acetate Test

Test for Alkaloids: 1. Dragendorff’s Test 2. Mayer’s Test 3. Hager’s Test 4. Wagner’s Test

Test for Tannins and Phenolic compounds: 1. 5% Ferric Chloride 2. Dilute Iodine Solution 3. Lead acetate solution 4. Dilute Potassium permanganate solution

### Cells, viral strain and culture conditions

TZM-bl (recombinant HeLa cells expressing high levels CD4 receptor, CXCR4 and CCR5 co-receptors) and PM1 cells (Clonal derivative of HUT 78) were obtained from the National Institutes of Health AIDS Research and Reference Reagent Program (NIH ARRRP). The HEC-1A (human endometrial adenocarcinoma) cell line was kindly provided by Dr. R. Fichorova (Associate Professor, Brigham and Women’s Hospital, Boston, USA) and National Institute of Virology, Pune, respectively.

The TZM-bl cells were maintained in Dulbecco’s modified Eagle’s medium (DMEM, Sigma-Aldrich, USA) and PM1 and HEC-1A cells in RPMI-1640 (Sigma-Aldrich, USA), supplemented with 10% heat inactivated fetal bovine serum (FBS, Moregate Biotech, Australia) and standard antibiotic-antimycotic cocktail.

The laboratory adapted HIV-1 strains [HIV-1IIIB (X4, subtype B), HIV-1Ada5 (R5, subtype B)] and the primary isolate HIV-1UG070 (X4, Subtype D) were procured from National Institutes of Health-AIDS Research and Reference Reagent Program, while the Indian isolate HIV-1VB59 (R5, subtype C) was obtained from the National AIDS Research Institute (NARI), Pune. Phytohemagglutinin-P (5 μg/ml, Sigma Aldrich, USA) activated peripheral blood mononuclear cells (PBMC) derived from healthy donors were used for the growth of all the viral strains. HIV-1 p24 antigen detection kit (Vironostika HIV-1 Antigen, Netherlands) was used to determine the virus production in cell culture supernatants. Samples of viral culture supernatants free form cells were obtained by centrifugation and further filtered and finally stored at -70oC for further use. Spearman Karber formula was used to ascertain the 50% tissue culture infectivity dose (TCID50) of each virus stock in both TZM-bl and PM1 cells [68].

### Anti HIV1 assays

#### Determination of cytotoxicity in the uninfected TZM-bl and PM1 cell lines

The cytotoxicity of the extracts was determined in uninfected TZM-bl cells using colorimetric assay that measures the reduction of a yellow 3-(4,5-dimethythiazol-2-yl)-2,5-diphenyl tetrazolium bromide (MTT) by mitochondrial succinate dehydrogenase (Sigma Aldrich, USA) [69]. Briefly, two-fold dilutions of the extracts were prepared, added to 96 well plates pre-seeded with TZM-bl cells (10,000 cells/well) in quadruplicate and incubated for 48 h at 37 °C. The MTT (20 μl, 5 mg/ml) solution was added and the plates were incubated further for 4 h. The supernatant was removed, 200 μl of DMSO was added, the plates were incubated for 1 h and the absorbance was read at 550 nm and 630 nm. The percent viability was calculated by comparing cell viability in the absence of extract using following formula and the results were expressed as CC_50_ (50% cytotoxic concentration).

$$ \%\mathrm{Cell}\ \mathrm{Viability}=\left[\mathrm{OD}\ \mathrm{test}\ \mathrm{extract}/\mathrm{Average}\ \mathrm{OD}\ \mathrm{control}\right]\times 100 $$

The cytotoxicity in uninfected PM1 cells of all the extracts was determined in a similar manner by using a similar dilution scheme and procedure as mentioned above for TZM-bl cells. The cell viability was determined by the trypan blue dye exclusion assay (Sigma Aldrich, USA) and the results were expressed as CC_50_ [70].

#### Preliminary screening for anti-HIV1 activity against laboratory adapted strains in TZM-bl cell lines

The anti-HIV1 activity was tested against Cell-free (CF) and Cell-associated (CA) X4 tropic (HIV-1 _IIIB_) and R5 tropic (HIV-1_Ada5_) laboratory adapted strains in TZM-b1 cell lines.

In cell free assay, the viral stocks (400 TCID_50_) were pre-treated in duplicate with sub toxic concentrations of the extracts/fractions for 1 h, at 37 °C prior to addition onto the TZM-bl cells (10,000 cells/well). While in cell-associated assay, the cells (10,000 cells/well) were pre-infected with the viral stocks (400 TCID_50_) for 2 h at 37 °C before exposure to the extracts/fractions [71]. After 48 h, the supernatant was collected and luciferase activity was determined using Britelite plus (Perkin Elmer, USA). Dextran Sulphate (Sigma Aldrich, USA) and Azidothymidine (AZT, CIPLA, India) were used as positive controls for cell free and cell associated assays respectively. The results were expressed as IC_50_ (50% inhibitory concentration), IC_80_ (80% inhibitory concentration) and Therapeutic Index (TI=CC_50_/IC_50_).

#### Confirmation of anti-HIV1 activity against primary isolates in TZM-bl and PM1 cell lines

The anti-HIV1 activity was tested against Cell-free (CF) and Cell-associated (CA) X4 tropic (HIV-1 _UG070_) and R5 tropic (HIV-1 _VB59_) primary isolates in TZM-b1 and PM1 cell lines.

The procedure for anti-HIV1 activity against primary isolates in TZM-bl cell lines followed was same as mentioned above for anti-HIV1 activity against laboratory adapted strains. The results were expressed as percentage inhibition calculated using following equation

The results were expressed as IC_80_ (80% inhibitory concentration).

The anti-HIV activity against primary isolates was also evaluated in PM1 cell lines using 24-well plate (Corning, USA). In the cell free assay, 20 TCID_50_ the viral stock (HIV-1_UG070_ and HIV-1_VB59_) was pre-treated with sub toxic concentrations of the extracts/fractions, before addition onto the cells (5x10^4^cells/well). Whereas, in the cell associated assay, the PM1 cells (5x10^4^cells/well) were pre-infected with 20 TCID_50_ of the viral stock and then exposed to the extracts/fractions [72]. The virus growth was monitored by Vironostika®p24 antigen ELISA (Biomerieux, France). Dextran sulphate and AZT were used as positive controls for cell free and cell associated assays respectively. The percent inhibition was calculated by comparing activity in absence of the extracts/fractions/control drug using the formula mentioned above and the results were expressed as IC_80._

### Toxicity testing using Transwell epithelial model

#### Cytotoxicity assay

The toxicity of selected plant extract was determined in HEC-1A using similar protocol as described for TZM-bl cells, only with a difference of the read out system, i.e. LDH cytotoxicity detection kit (Roche Diagnostics, Germany).

#### Determination of epithelial integrity in Transwell dual-chamber system

The epithelial integrity was determined as described by Gali et al.*,* [73]. Briefly, HEC-1A cells (1 × 10^5^/100 μl) were cultured for 7 days on the apical chamber of a Laminin coated dual-chamber Transwell® system (growth area: 0.3cm^2^, pore size: 3.0 μm) (Corning Costar Corp, USA). After 7 days incubation, two-fold serial dilutions of test preparations (100 μl) were added on to the HEC-1A cells and incubated for 24 h (37 °C, 5% CO_2_). The test preparations were removed and 100 μl of a 1/20 dilution of yellow-green fluorescent microspheres (FluoSpheres® sulphate microspheres, Molecular Probes Europe NV, Netherlands) were added in the apical chamber. After 24 h, 100 μl of medium was harvested from the basal chamber and the fluorescence was measured using a fluorometer (Perkin Elmer, USA). Untreated HEC-1A cells and 1% Nonoxynol-9 were used as controls for measuring percent transmission.

## Results

### Preliminary phytochemical investigation

The preliminary phytochemical evaluation of plant extracts for the presence of steriods, flavanoids, alkaloids, saponins, tannins and phenolic acids was done for 10 plants extracts from 8 different families. Steroids were not present in *T. procumbens, M. philippinensis, R. centifolia* and *A. squamosa* extracts and the Saponins in *T. procumbens* and *A. reticulate* extract*.* Only flavanoids was present in *T. procumbens* extract while it was not present in *S. potaotorum* extract (Table 3).

### Determination of cytotoxicity in TZM-bl and PM1 cell lines

Six methanolic extracts, two aqueous extracts and two hydroalcoholic extracts of 10 medicinal plants were examined for their ability to inhibit HIV-1 entry and replication. The in vitro toxicity of these extracts to TZM-bl cells was investigated by MTT assay. Methanolic extracts of, *A. aspera*, hydroalcoholic extract of *A. squamosa* and water extract of *F. benghalensis* tested were relatively non-toxic to TZM-bl cells at a CC_50_ value between 51 and 72 μg/ml. The CC_50_ values of other extracts such as methanolic extract of, *R. centifolia*, *S. potatorum* and aqueous extract of *F. infectoria* were found to be comparatively higher ranging between 118 and 147 μg/ml. However, methanolic extract of *A. reticulate* was found to be toxic at a very low concentration (CC_50_ = 11 μg/ml) as compared to the other extracts (Tables 4 and 5).

**Table 4:** Inhibitory concentrations and therapeutic index of plant extracts against Laboratory adapted HIV-1_IIIB_ and HIV-1_Ada5_ strains in TZM-bl cell lines

Sr. No.	Plant Extract	CC_50_ (μg/ml)	IC50				IC80				Therapeutic Index			
			CF		CA		CF		CA		CF		CA	
			IIIB	Ada5	IIIB	Ada5	IIIB	Ada5	IIIB	Ada5	IIIB	Ada5	IIIB	Ada5
1	*A. lebbeck*	203	No activity											
2	*T. procumbens*	62	No activity											
3	*A. aspera*	69	8.3	2.3	4.1	28.4	18	21	26	35	14	35	13	3
4	*F. benghalensis*	72	6	6	5.2	2.25	9.6	9.6	8.32	3.6	12	12	14	32
5	*M. philippinensis*	71	No activity											
6	*R. centifolia*	132	13.6	24.8	51.9	75.4	30.4	45.2	96.1	118	5	6	1	1
7	*S. potatorum*	124	4.97	3.65	18.51	17.67	29.17	35.89	79.35	78.43	10	24	8	7
8	*A. reticulata*	11	No activity											
9	*F. infectoria*	147	1.18	2.97	4.97	87.38	4.24	8.6	52.49	> 125	189	49	27	2
10	*A. squamosa*	51	No activity		23	11.3	No activity		27	20	2	4	2	3

CC_50−_50% cytotoxic concentration, IC_50_–50% inhibitory concentration

IC_80−_80% inhibitory concentration, CF- Cell Free, CA- Cell Associated

**Table 5:** Inhibitory concentrations of plant extracts against Primary isolates HIV-1UG070 and HIV-1VB59 in TZM-bl and PM1 cell lines

Extracts/ Fractions/Controls		TZM-bl assay					PM-1 assay				
		CC_50_ (μg/ml)	IC_80_(μg/ml)				CC_50_ (μg/ml)	IC_80_ (μg/ml)			
			CF HIV-1		CA HIV-1			CF HIV-1		CA HIV-1	
			UG 070	VB 59	UG 070	VB 59		UG 070	VB 59	UG 070	VB 59
*A. aspera*		69	4.8	< 19.53	26	53	10.7	2.6	8.4	1	1.6
*F. benghalensis*		72	< 78	< 156	< 156	< 78	2.9	3.5	2.9	NA	NA
*R. centifolia*		132	17	33.5	60.5	> 125	20	3.6	6.8	2.2	9
*S. potatorum*		124	< 31.25	80	60	105	46	29	NA	6	8.3
*F. infectoria*		147	18	22	42	73	28	2	29	NA	NA
*A. squamosa*		51	27	27	25	26	15.12	NA	NA	0.8	0.8
Control	DS	5553	6.43	4.5	–	–	4978	9.18	16.54	–	–
	AZT	782	–	–	8.01	18.70	998.5	–	–	8244.35	12,079.33

*CC*_*50*_ 50% cytotoxic concentration, *IC*_*80*_ 80% inhibitory concentration, *DS* Dextran Sulphate, *AZT* Azidothymidine, *CF* Cell Free, *CA* Cell Associated

Legend: The plant extracts showing 80% inhibition of HIV-1 primary isolates (UG070 & VB59) in TZM-bl cell line by cell free and cell associated assay with positive controls dextran sulphate and azidothymidine respectively

Cytotoxicity of plant extracts, *A. aspera, F. benghalensis, R. centifolia, S. potatorum, F. infectoria* and *A. squamosa* showing activity in preliminary anti-HIV-1 assay was carried out in PM1 cells using trypan blue dye exclusion assay. The 50% cytotoxicity was observed at a concentrations ranging from 2.9–46 μg/ml. Aqueous extract of *F. benghalensis* was toxic at a very low concentration as compared to other extracts (Table 5).

### Preliminary screening for anti-HIV1 activity against laboratory adapted strains in TZM-bl cell lines

Plant extracts of *A. aspera*, *F. benghalensis*, *R. centifolia*, *S. potatorum*, *F. infectoria* and *A. squamosa* showed inhibition of HIV-1_IIIB_ and HIV-1_Ada5_ laboratory adapted strains in both cell free and cell associated assays. Aqueous extract of *F. infectoria* revealed significant activity against the laboratory adapted strains with estimated IC_80_ in the range of 4.24–125 μg/ml giving TI of 189, 49 and 27 in cell free HIV-1_IIIB_, HIV-1_Ada5_ and cell associated HIV-1_IIIB_ respectively. This was followed by methanolic extract of *A. aspera* which showed activity with preliminary IC_80_ in the range of 18–35 μg/ml giving TI of 14, 35 and 13 in cell free HIV-1_IIIB_, HIV-1_Ada5_ and cell associated HIV-1_IIIB_ respectively. Aqueous extract of *F. benghalensis* exhibited activity in both laboratory adapted strains with estimated IC_80_ in the range of 18–35 μg/ml giving TI between12–32. Methanolic extract of *S. potatorum* showed activity with preliminary IC_80_ in the range of 29.17–79.35 μg/ml giving estimated TI of 24 in cell free HIV-1_Ada5_ strain. Methanolic extract of *R. centifolia* and hydroalcoholic extract of *A. squamosa* displayed very low activity (estimated TI in the range of 1–6). Hydroalcoholic extract of *A. lebbeck*, methanolic extract of *T. procumbens*, *M. philippinensis* and *A. reticulate* did not demonstrate any activity against cell free and cell-associated laboratory adapted HIV-1 strains.

### Confirmation of anti-HIV activity against primary isolates in TZM-bl and PM1 cell lines

The plant extracts showing activity in preliminary screening against laboratory adapted strains were further screened both cell free and cell associated assays against primary isolates HIV-1_UG070_ and HIV-1_VB59_ in TZM-b1 and PM1 cell lines for confirmation of their anti-HIV1 activity.

In TZM-b1 cell lines methanolic extract of *A. aspera* and hydroalcoholic extract of *A. squamosa* exhibited a very good activity with lowest estimated IC_80_ of 4.8–53 μg/ml and 25–27 μg/ml respectively against primary isolates of HIV-1 strains. This was followed by aqueous extract of *F. infectoria*, methanolic extract of *S. potatorum* and *R. centifolia* and aqueous extract of *F. benghalensis* with preliminary IC_80_ in the range of 18–73 μg/ml, < 31.25–105 μg/ml, 17- > 125 μg/ml, and < 78- < 156 μg/ml respectively (Table 5).

In PM1 cell lines, methanolic extract of *A. aspera* and *R. centifolia* showed activity with estimated IC_80_ ranging 1–9 μg/ml. Aqueous extract of *F. benghalensis* exhibited activity at preliminary IC_80_ of 2.9–3.5 μg/ml in cell free assay. Methanolic extract of *S. potatorum* exhibited activity at IC_80_ of 6–29 μg/ml except for HIV-1_VB59_ cell free assay. The hydroalcoholic extract of *A. squamosa* showed very good activity at lower concentration 0.8 μg/ml in cell associated assay. Aqueous extract of *F. infectoria* showed activity in cell free assay at a concentration ranging 2–29 μg/ml (Table 5). The representative dose response bar graphs for *A. aspera and R. centifolia*, in cell free and cell associated assays for TZMb1 and PM1 are given in Additional file 1: (Figures S1 to S5).

### Toxicity testing using Transwell epithelial model

**Fig. 1:**
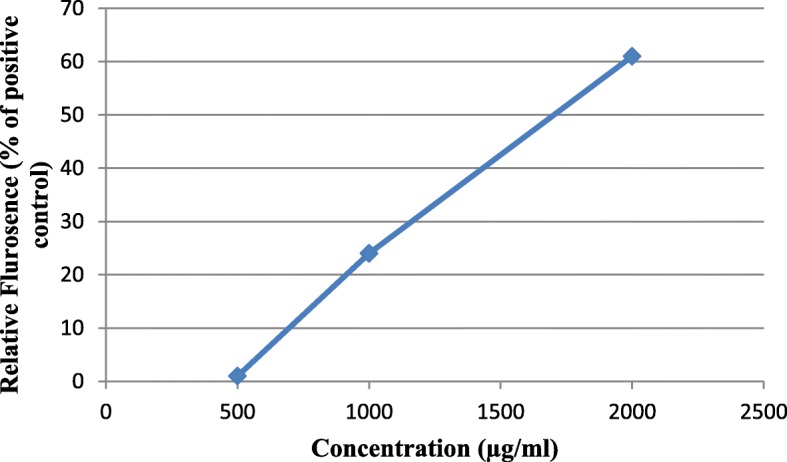
Plot of Relative Fluroscence (%) Vs Concentration (μg/ml) determining epithelial integrity of *R. centifolia* by measuring permeability to FluoSpheres using the Dual-Transwell Epithelial Model

Amongst the three extracts which showed highest activity, methanolic extract of *R. centifolia* exhibited least toxicity both inTZM-b1 and PM1 cell lines, therefore it was further tested for in vitro activity against HEC-1A cells and demonstrated minimal cytotoxicity with CC_50_ of 1443 μg/ml. Epithelial integrity of HEC-1A in Transwell dual-chamber system was maintained with only 1% relative fluorescence (percent of positive control) detected after treatment with 500 μg/ml of methanolic extract of *R. centifolia.* At higher concentration of 1000 μg/ml integrity was affected with 24% leaked fluorescence relative to positive control (Fig. 1).

## Discussion

Natural products continue to be major sources of innovative therapeutic agents for treatment of infectious diseases, and their exploration has been one of the most successful strategies for the discovery of medicines. The development of new microbicides as preventive interventions is a promising area in AIDS research [3]. They could be valuable addition in prevention of sexual transmission of HIV-1 and could be an important way to reduce the number of cases of HIV infection globally [74, 75]. Currently available anti-HIV drugs are chemically synthesized and are often limited by side effects and emergence of drug resistance [76].

In order to find such potential anti-HIV agents from natural sources, ten traditional medicinal plants from India were studied for their inhibitory effects against laboratory adapted strains HIV-1 IIIB, HIV-1Ada5 and primary isolates HIV-1UGO70, HIV-1VB59 in TZM-b1 and primary isolates HIV-1UGO70, HIV-1VB59 in PM1 cell lines. HIV viruses can spread in the body via either a cell-free (virus floating free in plasma) mode or a cell associated (virus particles that remain attached to or within the host cell after replication) mode involving direct cell-cell contact. Hence all the selected plant extracts were evaluated to depict their mechanism of action, whether they will act as an entry inhibitor or at the HIV replication stage [77].

The selected plant extracts were subjected to high throughput (cost-effective, quick and reproducible) TZM-bl assay model which is useful for preliminary screening allowing screening of large number of products against HIV [70, 78]. The results presented here indicate that the methanolic extracts of aerial parts of *A. aspera,* leaves of *R. centifolia* and seeds of *S. potatorum* and aqueous extract of leaves of *F. benghalensis* and *F. infectoria* possess anti-HIV properties of therapeutic interest inhibiting HIV-1 virus at an estimated TI of 3–35, 1–6, 7–24,12–32, and 2–189 respectively against laboratory adapted strains and at a very low preliminary IC_80_ ranging from 4.8–26, 17–125, 31–105, 78–156 and 18–73 μg/ml respectively against primary isolates using TZM-b1 assay.

The lead extracts were also confirmed for anti-HIV activity in PM1 cell line which supports persistent HIV-1 infection [72]. The PM1 cell line have been reported to be comparable to peripheral blood mononuclear cells (PBMCs) for culturing of any of the HIV-1 strains and subtypes and thus provide a valuable research tool for studying new anti-HIV therapies [79]. This cell line has been previously used for studying the anti-HIV1 properties of the polyherbal cream Basant [80]. Hence PM-1 was used for confirming the anti-HIV activity of the methanolic extracts of aerial parts of *A. aspera* and leaves of *R. centifolia* which showed anti-HIV activity (IC_80_) ranging between 1 and 8.4 and 2.2–6.8 μg/ml respectively. These extracts may potentially inhibit the entry and also inhibit HIV-1 replication if the virus enters the vaginal cells. However further work on more replicates and wider concentration range studies are required for confirmation. Future studies on PBMCs for qualifying the results should also be considered.

Our earlier work has shown that methanolic extract of *R. centifolia* has also shown activity against four strains of *N. gonorrhoeae* [81]. It substantially lacks cytotoxicity even at high concentrations (CC_50_ greater than 1 mg/ml) when tested in vitro on HEC-1A cell line (endometrial origin) and maintained its epithelial integrity when studied in Transwell model at concentrations up to 500 μg/ml thus showing potential for investigating it further as candidate anti-HIV microbicide.

As per the literature these extracts have not been further analyzed chemically, although the active components such as oleanolic acid and pomolic acid isolated from *Rosa wodsii* leaves the other species of *Rosa,* have been reported to inhibit HIV replication in acutely infected H9 cell growth at IC_50_ of 40 μg/ml [56, 57]. The literature indicates the phytosteroids, polyphenols and saponins present in the methanolic leaf extract of *A. aspera* are responsible for its anti-fertility effect [66] and methanolic root extract possess anti-herpes virus activity at EC_50_ of 64.4 μg/ml for HSV-1 and 72.8 μg/ml for HSV-2 [82]

The other selected medicinal plants extract showed anti-HIV activity against at least any one of the assay model except for hydroalcoholic extract of whole pods of *A. lebbeck*, methanolic extract of aerial parts of *T. procumbens*, methanolic extract of leaves of *M. philippinenis* and methanolic extract of leaves of *A. reticulate*, they were incapable of showing anti-HIV1 activity against cell free and cell associated HIV-1_IIIB_, HIV-1_Ada5_ laboratory adapted strains and HIV-1_UG070_, HIV-1_VB59_ primary isolates in TZM-b1 and PM1 cell lines. It’s worth mentioning that these plants were selected on basis of their sub species showing activity against other strains and primary isolates of HIV and the same species having contraceptive and activity related to this infectious disease. The inactivity of these plants against our test strains and primary isolates of HIV does not prove that they do not possess anti-HIV1 activity. These plants can be taken further for the activity against other strains and primary isolates of HIV virus using other anti-HIV assays.

Some plants extract such as *F. benghalensis, S. potatorum* and *F. infectoria* showed moderate to mild anti–HIV1 activity. These plants extracts had variable activities across the assays presented in this study where the extract exhibited inhibition of one strain of the primary isolates in one assay but did not inhibit the same primary isolates in another assay model. Aqueous extract of leaves of *F. benghalensis* showed anti-HIV1 activity against all HIV-1 laboratory adapted strains and primary isolates using TZM-b1 assay (TI: 12–32, IC_80_: 78–156 μg/ml) but did not inhibit cell associated primary isolates in PM1 assay. Methanolic extract of seeds of *S. potatorum* showed anti-HIV1 activity against all HIV-1 laboratory adapted strains and primary isolates using TZM-b1 assay (TI: 7–24, IC_80_: 31.25–105 μg/ml) but was not capable of inhibiting cell free primary isolate HIV-1_VB59_ in PM1 assay. Aqueous extract of leaves of *F. infectoria* showed anti-HIV1 activity against all HIV-1 laboratory adapted strains and primary isolates using TZM-b1 assay (TI: 2–189, IC_80_: 18–73 μg/ml) but did not inhibit cell associated primary isolates in PM1 assay. Hence these extracts may not altogether be classified as extracts not having anti-HIV1 inhibitory potential.

Hydroalcoholic extract of seeds of *A. squamosa* exhibited activity against all HIV-1 laboratory adapted strains and primary isolates using TZM-b1 assay (TI: 2–4, IC_80_: 26–27 μg/ml) but was not capable of inhibiting cell free primary isolates in PM1 assay. This plant extract has a some potential to be explored further and may be used supplementary as a replication inhibitor.

## Conclusion

To conclude the study, out of 10 plants screened for anti-HIV activity using TZM-b1 and PM1 assays, methanolic extracts of aerial parts of *A. aspera* and leaves of *R. centifolia* has prospective anti-HIV1 potential as an entry and replication inhibitors. Hence these experimental moieties may have favourable implications on the prevention or management of HIV/AIDS. Additionally methanolic extract of leaves of *R. centifolia* have shown good safety and maintained the epithelial integrity on HEC-1A cells. Plant extracts are complex mixtures of many compounds. Some compounds may mask the anti-HIV1 potential of plant extract due to their cytotoxicity. Therefore our next step would be isolating the phytoconstituents and increasing the chances to find active anti HIV1 compounds with low cytotoxicity.

## Supplementary information

**Additional file 1: Figure S1.** Plot of % HIV inhibition Vs Concentration (μg/ml) in TZMb1 (primary isolates) Cell free (CF) and Cell associated (CA) assay for *R. centifolia.***Figure S2.** Plot of % HIV inhibition Vs Concentration (μg/ml) in TZMb1 (primary isolates) Cell free assay for *A. aspera*. **Figure S3.** Plot of % HIV inhibition Vs Concentration (μg/ml) in TZMb1 (primary isolates) Cell associated assay for *A. aspera*. **Figure S4.** Plot of % HIV inhibition Vs Concentration (μg/ml) in PM1 Cell free assay for *R. centifolia and A. aspera*. **Figure S5.** Plot of % HIV inhibition Vs Concentration (μg/ml) in PM1 Cell associated assay for *R. centifolia* and *A. aspera*.

## Data Availability

The datasets used and/or analysed during the current study available from the corresponding author on reasonable request.

## Abbreviations

AIDS: Acquired Immunodeficiency Syndrome
CA: Cell-associated
CC_50_: 50% cytotoxic concentration
CF: Cell-free
DMEM: Dulbecco’s modified Eagle’s medium
DMSO: Dimethyl Sulphoxide
FBS: Fetal Bovine Serum
HAART: Highly Active Antiretroviral Therapy
HEC-1A: Human Endometrial Adenocarcinoma
HIV: Human Immunodeficiency Virus
HSV: Herpes Simplex Virus
IC_80_: 80% inhibitory concentration
LDH: Lactate Dehydrogenase
MTT: 3-(4,5-dimethythiazol-2-yl)-2,5-diphenyl tetrazolium bromide
OD: Optical Density
PBMC: Peripheral Blood Mononuclear Cells
PHA P: Phytohemagglutinin P
RPMI-1640: Roswell Park Memorial Institute1640 Medium
TCID_50_: Median Tissue Culture Infectious Dose
TI: Therapeutic Index

## References

[1] United Nations Programme on HIV/AIDS (UNAIDS), UNAIDS data 2019. Available online: https://www.unaids.org/en/resources/fact-sheethttps://www.hiv.gov/hiv-basics/overview/data-and-trends/global-statistics Accessed Oct 2019.

[2] Rege A, Ambaye RY, Deshmukh RA (2010). *In-vitro* testing of anti-HIV activity of some medicinal plants. Indian J Nat Prod Resour.

[3] D'Cruz OJ, Uckun FM (2004). Clinical development of microbicides for the prevention of HIV infection. Curr Pharm Des.

[4] Gupta RS, Kachhawa JB, Chaudhary R (2004). Antifertility effects of methanolic pod extract of *Albizia lebbeck* (L.) Benth in male rats. Asian J Androl.

[5] Karuppannan K, Subramanian D, Priyadharshini VS (2013). Phytopharmacological properties of *Albizia* species: a review. Int J Pharm Pharm Sci.

[6] Salahdeen HM, Yemitan OK, Alada AR (2004). An effect of aqueous leaf extract of *Tridax procumbens* on blood pressure and heart rate in rats. Afr J Biomed Res.

[7] Mahato RB, Chaudhary RP (2005). Ethnomedicinal study and antibacterial activities of selected plants of Palpa district, Nepal. Sci World.

[8] Saxena VK, Albert S (2005). b-Sitosterol-3-O-b-D-xylopyranoside from the flowers of *Tridax procumbens* Linn. J Chem Sci.

[9] Vilwanathan R, Shivashangari KS, Devak T (2005). Hepatoprotective activity of *Tridax procumbens* against d galactosamine/lipopolysaccharide-induced hepatitis in rats. J Ethnopharmacol.

[10] Bhagwat DA, Killedar SG, Adnaik AS (2008). Anti-diabetic activity of leaf extract of *Tridax procumbens*. Intnl J Green Pharma.

[11] Oladunmoye MK, Nutan Modi M, Dezzutti CS, Kulshreshtha S, Rawat A, Srivastava S (2006). Immunomodulatory effects of ethanolic extract of *Tridax procumbens* on swiss albino rats orogastrically dosed with *Pseudomonas aeruginosa* (NCIB 950). Int J Tropical Med.

[12] Vishnupriya P, Radhika K, Sivakumar R, Sri Ramchandra M, Prameela Devi V, Rao S (2011). Evaluation of anticancer activity of *T. procumbens* flower extracts on PC3 cell lines. Int J Adv Pharm Sci.

[13] Jayakumar T, Sridhar MP, Bharathprasad TR, Ilayaraja MG, S Balasubramanian MP. (2009). Experimental studies of *Achyranthes aspera* (L) preventing nephrotoxicity induced by lead in albino rats. J Health Sci.

[14] Khanna AK, Chander R, Singh C, Srivastava AK, Kapoor NK (1992). Hypolipidemic activity of *Achyranthes aspera* L. in normal and triton induced hyperlipemic rats. Indian J Exp Biol.

[15] Chakraborty A (2002). Cancer chemo preventive activity of *Achyranthes aspera* leaves on Espstein bar virus activation and two stages mouse skin carcinogenesis. Cancer Lett.

[16] Mishra SH, Bafna AR (2004). Effect of methanol extract of *Achyranthes aspera* Linn. on rifampicin-induced hepatotoxicity in rats. Ars Pharm.

[17] Shibeshi W, Makonnen E, Zerihun L, Debella A (2006). Estrogenic activity in Ethanolic extract of *Bupleram marginatum*. J Pharmacol Afr Health Sci.

[18] Saravanan P, Ramasamy V, Shivakumar T (2008). Antimicrobial activity of leaf extracts of *Achyranthes aspera* L. Asian J Chem.

[19] Gayathri DS, Archanah A, Abiramasundari P, Priya V, Uma K, Abirami T (2009). Pharmacological activities of *Achyranthes aspera*, an overview. Indian J Nutr Diet.

[20] Barua CC, Talukdar A, Begum SA, Buragohain B, Roy JD, Borah RS, Lahkar M (2009). Antidepressant like effects of *Achyranthes aspera* L. animals models of depression. Pharmacol..

[21] Paul DD, De KM, Ali K, Chatterjee DK, Nandi DG (2010). Effects of various extracts from the roots of *Achyranthes aspera*. Contraception..

[22] Khan MT, Ahmad K, Alvi MN, Mansoor B, Asif SM, Khan FZ (2010). Antibacterial and irritant activities of organic solvent extracts of *Agave americana* Linn., *Albizia lebbeck* Benth., *Achyranthes aspera* Linn. and *Abutilon indicum* Linn. - a preliminary investigation. Pakistan J Zool.

[23] Gabhe S, Tatke P, Khan T (2006). Evaluation of the immunomodulatory activity of the methanol extract of *Ficus benghalensis* roots in rats. Indian J Pharm.

[24] Parekh J, Jadeja D, Chanda S (2005). Efficacy of aqueous and methanol extracts of some medicinal plants for potential antibacterial activity. Turk J Biol.

[25] Gayathri M, Krishnan K (2008). Antidiabetic and ameliorative potential of *Ficus bengalensis* bark extract in Streptozotocin induced diabetic rats. Indian J Clin Biochem.

[26] Shukla R, Gupta S, Gambhir JK, Prabhu KM, Murthy PS (2004). Antioxidant effect of aqueous extract of the bark of *Ficus bengalensis* in hypercholesterolaemic rabbits. J Ethnopharmacol.

[27] Taur DJ, Nirmal SA, Patil RY, Kharya MD (2007). Antistress and ant allergic effects of *Ficus bengalensis* bark in asthma. Nat Prod Res.

[28] Gupta AK, Dwivedi S, Sharma A, Lodhi GS (2013). Evaluation of antihyperlipidemic, hypoglycemic and antioxidant potential of *Ficus infectoria* methanolic extract in wistar rats. J Pharmacognosy Phytochemistry.

[29] Daikonya A, Katsuki S, Wu JB, Kitanaka S (2002). Anti-allergic agents from natural sources (4): anti-allergic activity of new phloroglucinol derivatives from *Mallotus philippinensis* (Euphorbiaceae). Chem Pharm Bull.

[30] Thakur SC, Thakur SS, Chaube SK, Singh SP (2005). An etheral extract of Kamala (*Mallotus philippinensis* (mull. Arg) lam.) seed induce adverse effects on reproductive parameters of female rats. Reprod Toxicol.

[31] Moorthy K, Srinivasan K, Subramanian C, Mohanasundari C, Palaniswamy M (2007). Phytochemical screening and antibacterial evaluation of stembark of *Mallotus philippinensis* var. Tomentosus. Afr J Biotechnol.

[32] Tanaka R, Nakata T, Yamaguchi C, Wada S, Yamada T, Tokuda H (2008). Potential anti-tumor-promoting activity of 3α-Hydroxy-D: a-friedooleanan2-one from the stem bark of *Mallotus philippinensis*. Planta Med.

[33] Arfan M, Hazrat K, Magdalena K (2009). Antioxidant activity of phenolic fractions of *Mallotus philippinensis* bark extract. J Food Sci.

[34] Ramakrishna S, Geetha KM, Bhaskargopal PVV, Kumar SRP, Madav CP, Umachandar L (2011). Effect of *Mallotus philippinensis* Muell-Arg leaves against hepatotoxicity of carbon tetrachloride in rats. Int J Pharm Sci Res.

[35] Khan M, Qureshi RA, Hussain M, Mehmood K, Khan RA (2013). Hexane soluble extract of *Mallotus philippinensis* (lam.) Muell. Arg. root possesses anti-leukaemic activity. Chem Cent J.

[36] Roshan R, Kulkarni SG, Tupe SP (2014). Antifungal dimeric Chalcone derivative Kamalachalcone E from *Mallotus philippinensis*. Nat Prod Res.

[37] Hassan A, Nafisa. An investigation of antimicrobial compounds for immunomodulating and anti-adhesion properties. Pak Res Repository. 2003.

[38] Sankaranand R (2011). Evaluation of antitussive activity of *Rosa centifolia*. IJPSR.

[39] Gupta RS, Kanwar M, Rehwani H, Verma SK, Dobhal MP (2006). Contraceptive efficacy of *Strychnos potatorum* seed extract in male albino rats. Asian J Exp Sci.

[40] Biswas S, Murugesan T, Sinha S, Maiti K, Gayen JR, Pal M, Sah BP (2002). Antidiarrhoeal activity of *Strychnos potatorum* seed extract in rats. Fitoterapia.

[41] Sanmugapriya E, Venkataraman S (2006). Studies on hepatoprotective and antioxidant actions of *Strychnos potatorum* Linn. seeds on CCl -induced acute hepatic injury in experimental rats. J Ethnopharmacol.

[42] Sanmugapriyaa E, Venkataraman S (2007). Antiulcerogenic potential of *Strychnos potatorum* Linn. seeds on Aspirin plus pyloric ligation-induced ulcers in experimental rats. Phytomedicine.

[43] Mallikharjuna PB, Seetharam YN (2009). *In vitro* antimicrobial screening of alkaloid fractions from *Strychnos potatorum*. J Chemother.

[44] Ekambaram S, Perumal SS, Venkataraman S (2010). Evaluation of antiarthritic activity of *Strychnos potatorum* Linn seeds in Freund's adjuvant induced arthritic rat model. BMC Complement Altern Med.

[45] Dhasarathan P, Theriappan P (2011). Evaluation of antidiabetic activity of *Strychnos potatorum* in alloxan induced diabetic rats. J Med Med Sci.

[46] Yuan SSF, Hl C, Chen HW, Yeh YT, Kao YH, Lin KH (2003). Annonacin, a mono-tetrahydrofuran acetogenin, arrests cancer cells at the G1 phase and causes cytotoxicity in a Bax- and caspase-3-related pathway. Life Sci.

[47] Baskar R, Rajeswari V, Sathish KT (2007). *In vitro* antioxidant studies in leaves of *Annona* species. Indian J Exp Biol.

[48] Thang TD, Kuo PC, Huang GJ, Hung NH, Huang BS, Yang ML (2013). Chemical constituents from the leaves of *Annona reticulata* and their inhibitory effects on NO production. Molecules.

[49] Wu YC, Hung YC, Chang FR, Cosentino M, Wang HK, Lee KH (1996). Identification of ent-16,17-dihydroxykauran-19-oicacid as an anti-HIV principle and isolation of the new diterpenoids, annosquamosins a and B from *Annona squamosa*. J Nat Prod.

[50] Khar A, Pardhasaradhi BVV, Reddy M, Ali Mubarak A, KumariLeela A (2004). Antitumour activity of *Annona squamosa* seed extracts is through the generation of free radicals and induction of apoptosis. Indian J Biochem Biophys.

[51] Mohamed (2008). Hepatoprotective activity of *Annona squamosa* Linn. on experimental animal model. Int J Appl Res Nat Prod.

[52] Jayshree P, Kumar V (2008). *Annona squamosa* L.: phytochemical analysis and antimicrobial screening. J Pharm Res.

[53] Johns (2011). Antimalarial alkaloids isolated from *Annona squamosa*. Phytopharmacology.

[54] Yadav DK, Singh N (2011). Anti-ulcer constituents of *Annona squamosa* twigs. Fitoterapia.

[55] Chandrashekar (2011). Isolation, characterizations and free radical scavenging activity of *Annona squamosa* leaf. J Pharm Res.

[56] Vlietinck AJ, De Bruyne T, Apers S, Pieters LA (1988). Plant-derived leading compounds for chemotherapy of human immunodeficiency virus (HIV) infection. Planta Med.

[57] Kashiwada Y, Wang HK, Nagao T, Kitanaka S, Yasuda I, Fujioka T (1998). Anti-AIDS agents. 30. Anti-HIV activity of oleanolic acid, pomolic acid, and structurally related triterpenoids. J Nat Prod.

[58] Yu YB, Park JC, Lee JH, Kim GE, Jo SK, Byun MW (1998). Screening of some plant extracts for inhibitory effects on HIV-1 and its essential enzymes. Korean J Pharmacol.

[59] Ramanathan T, Premanathan M, Kathiresan K, Nakashima H, Yamamole N (1999). Studies on some coastal plants for anti-HIV activity.

[60] Nakane H, Arisawa M, Fujita A, Koshimura S, Ono K (1991). Inhibition of HIV reverse transcriptase activity by some fluroglucinol derivatives. FEBS Lett.

[61] Yadav KN, Kadam PV, Patel JA, Patil MJ (2014). *Strychnos potatorum*: phytochemical and pharmacological review. Pharmacogn Rev.

[62] Singh KK, Parmar S, Tatke PA (2012). Contraceptive efficacy and safety of HerbOshield™ vaginal gel in rats. Contraception.

[63] Taylor RSL, Hudson JB, Manandhar NP, Towers GHN (1996). Antiviral activities of medicinal plants of southern Nepal. J Ethnopharmacol.

[64] Taylor RSL, Manandhar NP, Hudson JB, Towers GHN (1996). Antiviral activities of Nepalese medicinal plants. J Ethnopharmacol.

[65] Lederman MM, Offord RE, Hartley O (2006). Microbicides and other topical strategies to prevent vaginal transmission of HIV. Nat Rev Immunol.

[66] Workineh S, Eyasu M, Asfaw D, Legesse Z (2006). Phytochemical, contraceptive efficacy and safety evaluations of the methanolic leaves extract of *Achyranthes aspera* L. in rats. Pharmacologyonline..

[67] Harbourne JB (1998). Phytochemical methods. A guide to modern technique of plant analysis.

[68] Connick E, Campbell T, Schneider K, Wrin T (2003). Relationship between *in vitro* human immunodeficiency virus type 1 replication rate and virus load in plasma. J Virol.

[69] Mosmann T (1983). Rapid colorimetric assay for cellular growth and survival: application to proliferation and cytotoxicity assays. J Immunol Methods.

[70] Platt EJ, Wehrly K, Kuhmann SE, Chesebro B, Kabat D (1998). Effects of CCR5 and CD4 cell surface concentrations on infection by macrophage tropic isolates of human immunodeficiency virus type 1. J Virol.

[71] Verma A, Ranga U, Gupta S (2013). Extracts from *Acacia catechu* suppress HIV-1 replication by inhibiting the activities of the viral protease and tat. Virol J.

[72] Lusso P, Cocchi F, Balotta C, Markham P, Louie A, Farci P (1995). Growth of macrophage-tropic and primary human immunodeficiency virus type 1 (HIV-1) isolates in a unique CD4+ T-cell clone (PM1): failure to downregulate CD4 and to interfere with cell-line-tropic HIV-1. J Virol.

[73] Gali Y, Delezay O, Brouwers J, Addad N, Augustijns P, Bourlet T (2010). *In vitro* evaluation of viability, integrity, and inflammation in genital epithelia upon exposure to pharmaceutical excipients and candidate microbicides. Antimicrob Agents Chemother.

[74] Gaym A (2006). Microbicides-emerging essential pillars of comprehensive HIV/AIDS prevention. Ethiop Med J.

[75] Howett MK, Kuhl JP (2005). Microbicides for prevention of transmission of sexually transmitted diseases. Curr Pharm Des.

[76] De Clercq E (2002). New developments in anti HIV chemotherapy. Biochim Biophys Acta.

[77] Mothes W, Sherer N, Jing J, Zhong P (2010). Virus cell-to-cell transmission. J Virol.

[78] Wei X, Decker JM, Liu H, Zhang Z, Arani RB, Kilby JM (2002). Emergence of resistant human immunodeficiency virus type 1 in patients receiving fusion inhibitor (T-20) monotherapy. Antimicrob Agents Chemother.

[79] Herrera-Carrillo E, Paxton WA, Berkhout B (2014). The search for a T cell line for testing novel antiviral strategies against HIV-1 isolates of diverse receptor tropism and subtype origin. J Virol Methods.

[80] Maselko MB, Joshi RS, Prescott M, Talwar GP, Kulkarni S, et al. Basant, a Polyherbal topical Microbicide candidate inhibits different clades of both CCR5 and CXCR4 tropic, lab-adapted and primary isolates of Human Immunodeficiency Virus-1 *in vitro* infection. J Virol Antivir Res. 2014;3(4). 10.4172/2324-8955.1000131.

[81] Jadhav N, Kulkarni S, Mane A, Kulkarni R, Palshetker A, Singh K (2014). Antimicrobial activity of plant extracts against sexually transmitted pathogens. Nat Prod Res.

[82] Mukherjee H, Ojhaa D, Baga P, Chandel HS, Bhattacharyya S, Chatterjee TK (2013). Anti-herpes virus activities of *Achyranthes aspera*: an Indian ethnomedicine, and its triterpene acid. Microbiol Res.

