# Supplementary material for: In vitro anti-HIV activity of some Indian medicinal plant extracts
Source: BMC Complement Med Ther. 2020 Mar 6;20:69. doi: 10.1186/s12906-020-2816-x (PMC7076815; doi:10.1186/s12906-020-2816-x)
Supplement: Supplementary file 1 — Additional file 1: Figure S1. Plot of % HIV inhibition Vs Concentration (μg/ml) in TZMb1 (primary isolates) Cell free (CF) and Cell associated (CA) assay for R. centifolia.Figure S2. Plot of % HIV inhibition Vs Concentration (μg/ml) in TZMb1 (primary isolates) Cell free assay for A. aspera. Figure S3. Plot of % HIV inhibition Vs Concentration (μg/ml) in TZMb1 (primary isolates) Cell associated assay for A. aspera. Figure S4. Plot of % HIV inhibition Vs Concentration (μg/ml) in PM1 Cell free assay for R. centifolia and A. aspera. Figure S5. Plot of % HIV inhibition Vs Concentration (μg/ml) in PM1 Cell associated assay for R. centifolia and A. aspera. [file 12906_2020_2816_MOESM1_ESM.docx]

**Supplementary Data**

**Figure S1: Plot of % HIV inhibition Vs Concentration (µg/ml) in TZMb1 (primary isolates) Cell free (CF) and Cell associated (CA) assay for *R. centifolia.***

**Figure S2: Plot of % HIV inhibition Vs Concentration (µg/ml) in TZMb1 (primary isolates) Cell free assay for *A. aspera***

**Figure S3: Plot of % HIV inhibition Vs Concentration (µg/ml) in TZMb1 (primary isolates) Cell associated assay for *A. aspera***

**Figure S4: Plot of % HIV inhibition Vs Concentration (µg/ml) in PM1 Cell free assay for *R. centifolia and A. aspera***

**Figure S5 Plot of % HIV inhibition Vs Concentration (µg/ml) in PM1 Cell associated assay for *R. centifolia* and *A. aspera***
